# Suppression of Spry1 inhibits triple-negative breast cancer malignancy by decreasing EGF/EGFR mediated mesenchymal phenotype

**DOI:** 10.1038/srep23216

**Published:** 2016-03-15

**Authors:** Qing He, Hongyu Jing, Lucy Liaw, Lindsey Gower, Calvin Vary, Shucheng Hua, Xuehui Yang

**Affiliations:** 1 Center for Molecular Medicine, Maine Medical Center Research Institute, 81 Research Drive, Scarborough, ME, USA; 2 Department of Respiratory Medicine, The First Hospital of Jinlin University, Changchun, China

## Abstract

Sprouty (Spry) proteins have been implicated in cancer progression, but their role in triple-negative breast cancer (TNBC), a subtype of lethal and aggressive breast cancer, is unknown. Here, we reported that Spry1 is significantly expressed in TNBC specimen and MDA-MB-231 cells. To understand Spry1 regulation of signaling events controlling breast cancer phenotype, we used lentiviral delivery of human Spry1 shRNAs to suppress Spry1 expression in MDA-MB-231, an established TNBC cell line. Spry1 knockdown MDA-MB-231 cells displayed an epithelial phenotype with increased membrane E-cadherin expression. Knockdown of Spry1 impaired MDA-MB-231 cell migration, Matrigel invasion, and anchorage-dependent and -independent growth. Tumor xenografts originating from Spry1 knockdown MDA-MB-231 cells grew slower, had increased E-cadherin expression, and yielded fewer lung metastases compared to control. Furthermore, suppressing Spry1 in MDA-MB-231 cells impaired the induction of Snail and Slug expression by EGF, and this effect was associated with increased EGFR degradation and decreased EGFR/Grb2/Shp2/Gab1 signaling complex formation. The same phenotype was also observed in the TNBC cell line MDA-MB-157. Together, our results show that unlike in some tumors, where Spry may mediate tumor suppression, Spry1 plays a selective role in at least a subset of TNBC to promote the malignant phenotype via enhancing EGF-mediated mesenchymal phenotype.

Triple-negative breast cancer (TNBC) is an aggressive breast cancer subtype in which the tumor cells lack estrogen receptor and progesterone receptor expression, and do not overexpress human epidermal growth factor receptor 2 (HER2). It accounts for approximately 12–17% of all breast cancers^1^. Despite having higher rates of clinical response to pre-surgical chemotherapy, TNBC patients have high rate of recurrence and distant metastasis^2^. It is believed that epithelial to mesenchymal transition (EMT) is a defining step of cancer metastasis^3^, particularly in TNBC, the most lethal and aggressive subtype of breast cancer^4^,^5^,^6^.

EMT is characterized by loss of cell-cell adhesion due to down-regulation of junctional adhesion molecules such as E-cadherin. E-cadherin is regulated by transcriptional repressors including Snail, Slug, Zeb1, Zeb2 and Twist^7^,^8^,^9^,^10^,^11^. MAPK/ERK and PI3K/Akt signaling pathways induced by inappropriate activation of receptors such as EGFR, FGFR, PDGFR, have been shown to induce these transcription factors to promote EMT and cancer malignancy and metastasis^12^,^13^,^14^,^15^,^16^,^17^.

Sprouty (Spry) proteins are induced by and regulate multiple receptor tyrosine kinase (RTK) mediated MAPK/ERK signaling pathways, which play essential roles in cell proliferation, migration, differentiation and apoptosis. Specific roles of Spry proteins in tumor progression are still not being defined. Down-regulation of Spry1 and Spry2 occurs in multiple cancer types including prostate, liver, lung and breast cancers, suggesting a potential tumor suppressive effect in some contexts^18^,^19^,^20^. In contrast, Spry proteins promote the growth of various tumors harboring Raf or Ras mutations^21^,^22^,^23^, suggesting a role in malignancy. Indeed, suppression of Spry1 in rhabdomyosarcoma tumors with mutant Ras was sufficient to lead to complete tumor regression^24^. Mechanisms of Spry activity are likely to be dependent on tissue and cell context, and need to be determined for specific cancer subtypes.

In this study, we addressed the role of Spry1 in TNBC cell lines, where its function is not well understood. We demonstrate for the first time that suppression of Spry1 in these TNBC inhibits cell growth, invasion and metastasis by promoting mesenchymal to epithelial transition both *in vitro* and *in vivo*. Knockdown of Spry1 results in EGF mediated EGFR degradation, impairs the EGF/EGFR/Snail signaling, increases E-cadherin expression and cell-cell adhesion, and promotes these TNBC reversion from a mesenchymal to an epithelial phenotype. These observations indicate that endogenous Spry1 promotes tumor malignancy in at least a subset of TNBC, and may thus be a novel therapeutic target for this subset of TNBC breast cancers and may be beneficial for the cancer patients.

## Results

### Spry1 and Spry4 are expressed in human TNBC and elevated in the TNBC cell lines

**Figure 1:**
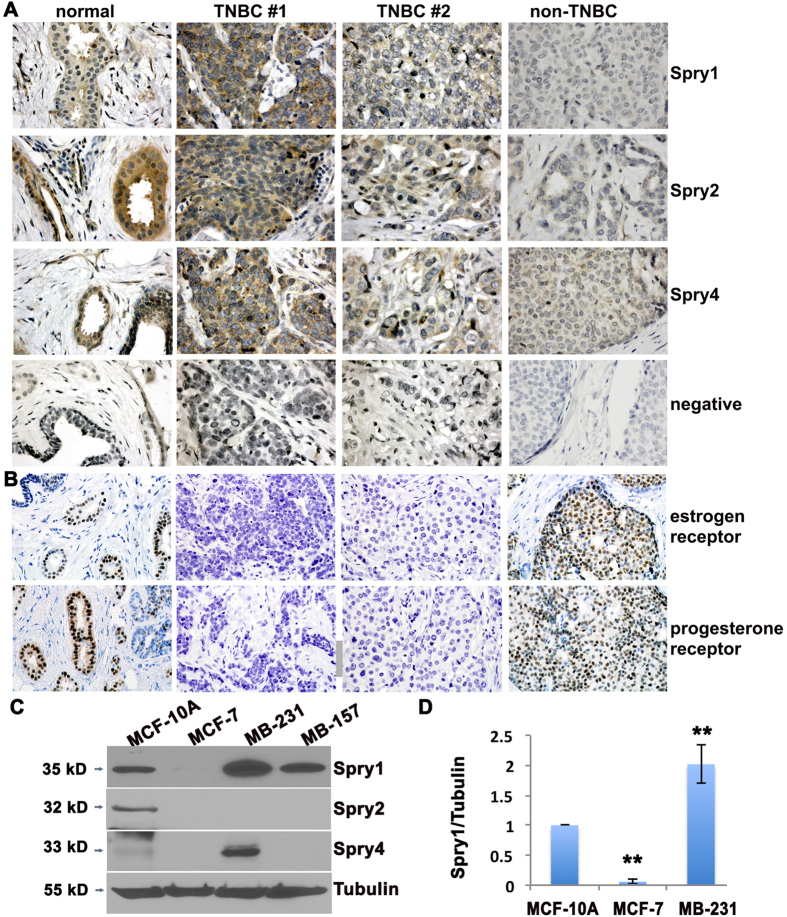
Spry1 is expressed in TNBC and in MDA-MB-231 TNBC cells. (**A**) Representative views of normal breast tissue versus biopsy from non-TNBC and TNBC tissues immunostained to detect Spry1, Spry2 and Spry4 (brown reaction product). Tissues were counterstained with hematoxylin. For negative controls (NEG), sections were incubated with normal rabbit IgG instead of specific antibody. (**B**) Sections were stained to detect the estrogen receptor or progesterone receptor, and counterstained with hematoxylin. (**C**) Immunoblotting to evaluate the expression of Spry proteins in the TNBC cell line MDA-MB-231 and MDA-MB-157 compared to the normal mammary epithelial cell line MCF-10A and the non-TNBC cell line MCF-7. The blots were stripped off and reused for probing of Tubulin. (**D**) Quantification of Spry1 protein levels normalized by tubulin levels from three independent experiments. **p < 0.05 relative to controls. Images are from original 400x magnification.

Immunohistochemistry was used to evaluate the protein levels of Sprys in a breast cancer tissue array composed of 17 TNBC, 6 non-TNBC, and available corresponding normal breast tissues. Baseline levels of Spry1, Spry2 and Spry4 were present at moderate levels in normal breast tissue (Fig. 1A), with slightly different patterns. Spry1 is weakly expressed in ductal epithelium but highly expressed in the surrounding basal cells and stromal fibroblasts, whereas Spry2 and Spry4 are predominantly expressed in ductal epithelial cells. The staining intensity was compared to that in different breast cancer samples, and staining was qualitatively scored as weak, moderate, or strong in comparison to the moderate levels in normal tissue. In agreement with a previous report, we observed low expression of Spry2 (5 out of 6) in non-TNBC, while the level of Spry4 was similar to normal breast tissue. There were lower levels of Spry2 in 7 out of 17 TNBC, with the other ten TNBC either having comparable (6/17) or increased (4/17) Spry2 levels. Interestingly, 11 out of 17 TNBC have equivalent, and 4 out of 17 TNBC have higher levels of Spry1 compared to normal breast tissues. For Spry4, 10 out of 17 TNBC have comparable levels to normal breast tissue, and 7 out of 17 TNBC have increased levels. The status of estrogen and progesterone receptor was verified by immunohistochemistry (Fig. 1B and data not shown). Spry levels in normal and malignant human breast tissues are summarized in supplementary Table S2.

We next examined levels of Sprys in TNBC cell line MDA-MB-231 and MDA-MB-157, a luminal breast cancer cell line MCF-7, and an immortalized human mammary epithelial cell line MCF-10A. Immunoblotting analysis showed moderate Spry1 and Spry2 protein in normal epithelial MCF-10A cells. By contrast, Spry1 was low in MCF-7 cells, higher in MDA-MB-231 and MDA-MB-157 cells, and Spry2 protein was undetectable in all three MCF-7, MDA-MB-231 and MDA-MB157 breast tumor cell lines. Spry4 has an inverse pattern, with low in MCF-10A and higher in MDA-MB-231 cells, (Fig. 1C,D). We also examined the expression of Sprys in two additional TNBC cell lines, Hs578T (HTB-126) and MDA-MB-468 cells, and found modest and minimal expression of Spry1 in Hs578T and MDA-MB-468 cells, respectively. Spry4 is expressed in Hs578T cells but undetectable in MDA-MB-468 cells, while Spry2 is undetectable in both cell lines (Supplementary Fig. S1). Collectively, Spry1 is expressed in all tested 4 TNBC cell lines albeit at varied levels, whereas Spry2 is undetectable.

### Knockdown of Spry1 inhibits MDA-MB-231 cell growth, migration and invasion

**Figure 2:**
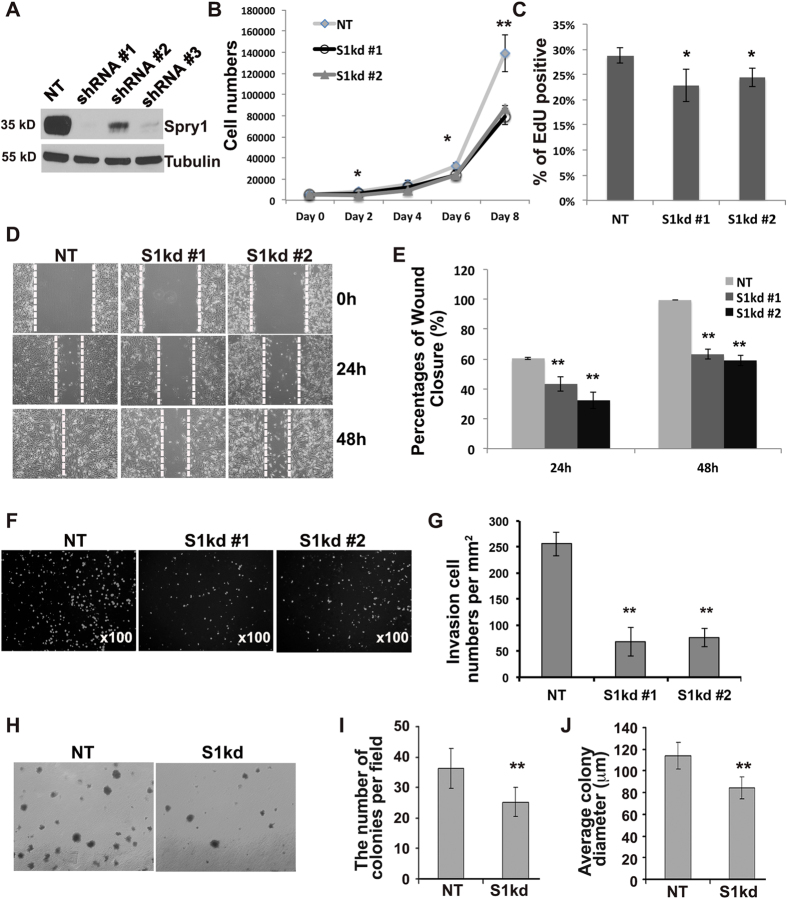
Knockdown of Spry1 decreases MDA-MB-231 cell growth, colony formation, migration and invasion. Human Spry1 shRNAs or non-targeting control shRNA lentiviruses were used to generate Spry1 knockdown (S1kd) or control non-targeting (NT) cell lines. (**A**) Lysates from NT or S1kd cells were immunoblotted to examine the effectiveness of shRNAs. The same blot was reused for probing Tubulin. (**B**) Cell growth curve analysis shows loss of Spry1 suppressed cell growth. (**C**) EdU labeling followed by FACS shows that knockdown of Spry1 decreased MDA-MB-231 cell proliferation. (**D**) Representative images from at least three independent scratch assays show suppressing Spry1 expression inhibited cell migration into the denuded area. (**E**) Quantification of cell migration capacity. (**F**) Representative images from at least three independent Matrigel invasion assays show that knockdown of Spry1 decreased MDA-MB-231 cell matrix invasion towards serum. (**G**) Quantification of the level of cell invasion. (**H**) Representative images from at least three independent soft-agar colony formation assays show that S1kd cells formed fewer and smaller colonies compared to NT cells. (**I,J**) Quantification of the colony number and size from the soft-agar assays. *p < 0.05, **p < 0.01 relative to controls.

**Figure 3:**
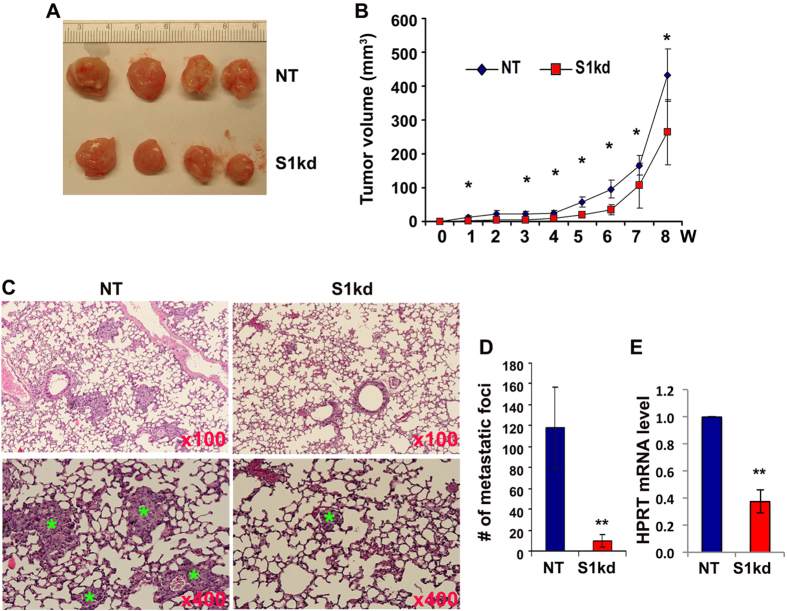
Knockdown of Spry1 decreases MDA-MB-231 tumor growth and lung metastasis *in vivo*. (**A**) Images of tumors harvested at 9 weeks after fat pad inoculation of 1 × 10^6^ S1kd or NT cells. (**B**) Tumor size was measured by calipers weekly, and the total volume was calculated. Shown is the tumor growth curve from inoculation of 1 × 10^6^ cells. (**C**) Representative H&E staining of lungs from 1 × 10^6^ dosage xenograft mice showing fewer and smaller cancer metastasis lesions in S1kd injected mice compared to NT injected mice. (**D**) Quantification of lung metastasis. (**E**) RT-qPCR analysis of human HRPT transcript versus total 18S rRNA transcripts in lungs from S1kd and NT cells injected mice, the relative mRNA level of human HRPT in S1kd tumors compare to NT tumors was presented. *p < 0.05, **p < 0.01 relative to controls. Four animals from each group were used for quantification analysis.

The high levels of Spry1in TNBC cell lines and in a subset of TNBC samples led us to hypothesize that Spry1 may have a role in promoting TNBC malignancy. To test this, we used shRNA lentiviruses to knock down Spry1 in the well-characterized MDA-MB-231 TNBC cell model. Three different human Spry1 shRNA lentiviral constructs were able to remarkably reduce endogenous Spry1 expression compared to non-targeting control (NT) (Fig. 2A). We then examined the behavior of tumor cells with suppressed Spry1 levels, using the cells treated with shRNA#1 (S1kd#1) and shRNA#2 (S1kd#2). Suppression of Spry1 corresponded to decreased cell growth in both populations (Fig. 2B). EdU incorporation followed by FACS analysis confirmed that the suppression of Spry1 decreased cell proliferation (Fig. 2C). Cell migration is a crucial cellular process that contributes to cancer malignancy. Therefore, we examined the migratory ability of Spry1 knockdown MDA-MB-231 cells. Confluent S1kd and NT cells were subjected to a scratch assay in the presence of mitomycin C to suppress cell proliferation, and cell migration into the denuded area was quantified. NT cells migrated into the denuded area within 24 h and completely closed the scratch after 48 h, whereas cells with suppressed Spry1 showed impaired cell migration, with the denuded area remaining after 48 h (Fig. 3D–E). We also tested cellular invasiveness using Matrigel coated transwell invasion assays, and observed that knocking down Spry1 inhibited MDA-MB-231 cell invasion (Fig. 3F–G). These results indicate that Spry1 is involved in cell mobility, invasion and metastasis, and its suppression can significantly suppress the MDA-MB-231 cell malignant phenotype.

Anchorage-independent growth is a key aspect of malignancy, and we tested the effects of decreased Spry1 on colony formation in soft agar. We found that knockdown of Spry1 led to decreased number and size of anchorage-independent colonies (Fig. 2H–J). We also observed that it was difficult to obtain stable cell transfectants with suppression of Spry1. We attribute this to a survival defect in cells lacking Spry1, since puromycin-resistant cells that grew out after a long latency (two weeks), has gradually restored Spry1 expression. Indeed, analysis of cell apoptosis revealed a slight but significant increase in annexin V staining in S1kd cells compared to NT cells (Supplementary Fig. S2).

### Knockdown of Spry1 inhibits MDA-MB-231 tumor growth and metastasis *in vivo*

We used an orthotopic xenograft model to analyze the role of Spry1 in TNBC tumor growth and metastasis *in vivo*. NT or S1kd MDA-MB-231 cells were injected into the mammary fat pads of eight-week old immunodeficient NOD/SCID mice at dosages of 1 × 10^6^, 1.5 × 10^6^ or 2 × 10^6^ cells per mouse. All injected mice developed tumors. However, S1kd tumors grew slower than NT tumors regardless of the number of cells injected (Fig. 3A,B and Supplementary Fig. S3), and had fewer spontaneous lung metastases than NT tumors as shown by histological staining (Fig. 3C,D). To get a more quantitative measurement of tumor cell metastasis to the lung, we used RT-qPCR to detect the levels of human HPRT transcript in the lung compared to total 18S rRNA, and confirmed the fewer metastases in S1kd tumor bearing mice than NT tumor bearing mice (Fig. 3E). We also performed immunohistochemistry to examine the expression of Spry1 in xenograft tumors, and confirmed a ~50–60% decrease of Spry1 expression in S1kd derived tumors compared to that in NT derived tumors (Supplementary Fig. S4).

### Knockdown of Spry1 induces MDA-MB-231 cell mesenchymal to epithelial transition

**Figure 4:**
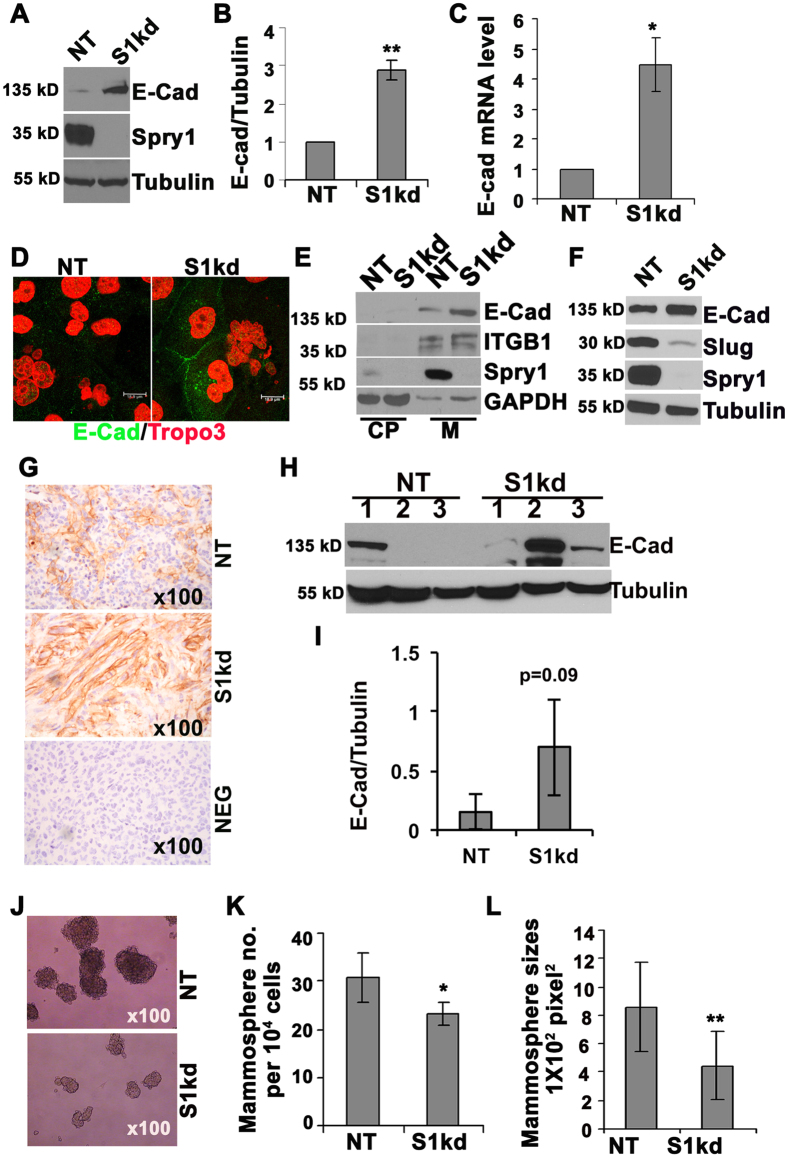
Knockdown of Spry1 induces an epithelial to mesenchymal transition in MDA-MB-231 cells. (**A**) Representative immunobotting from at least three independent experiments showing a reduction of endogenous Spry1 by shRNA lentiviruses transduction, and an increase of E-cadherin expression in S1kd compared NT cells. (**B**) Quantification of E-cadherin expression. (**C**) RT-qPCR shows increased E-cadherin mRNA level in S1kd compared to NT cells. Three independent experiments were used for quantification. (**D**) Immunofluorescence staining shows increased membrane associated E-cadherin in S1kd compared to NT cells. Representative images are from original 200x magnification. (**E**) Cell fractionation followed by immunoblotting shows that increased E-cadherin in S1kd cells was in the membrane fraction (M), and not in the cytoplasm (CP). (**F**) Representative immunoblotting from at least three independent experiments shows that the increased E-cadherin in S1kd cells was associated with decreased levels of the mesenchymal inducer Slug. (**G**) Representative E-cadherin staining of tumors showing increased E-cadherin signal in S1kd tumors compared to NT tumors. (**H**) Immunoblotting assay to evaluate E-cadherin protein levels in tumor extracts. (**I**) Quantification from immunoblotting (**H**). (**J**) Representative phase contrast images show that S1kd cells formed few and smaller mammospheres compared to NT cells. (**K,L**) Quantification of the number and the size of mammospheres from at least three independent experiments. *p < 0.05; **p < 0.01 relative to controls.

Normal mammary epithelial cells have a polygonal, epithelial morphology, characterized by high levels of junctional E-cadherin. During the process of tumorigenesis, epithelial cells lose E-cadherin and undergo an epithelial to mesenchymal transition (EMT). Highly malignant MDA-MB-231 cells have a fibroblastic, mesenchymal morphology, and lack E-cadherin. However, cells with Spry1 knockdown started to form epithelial-like patches (Supplementary Fig. S5), suggesting a reversion into a more epithelial phenotype. This morphological change was accompanied by a significant increase in E-cadherin protein (Fig. 4A,B) and mRNA (Fig. 4C). In addition, immunofluorescence confirmed increased cellular E-cadherin, including accumulation at cell junctions in S1kd cells (Fig. D). Cell fractionation analysis also confirmed increased membrane localization of E-cadherin in S1kd cells (Fig. 4E). Conversely, we were unable to detect N-cadherin protein in MDA-MB-231 cells (data not shown) in agreement with a previous report^25^. These results suggest that repression of Spry1 up-regulates the functional form of E-cadherin that promotes cell-cell adhesion. We also observed that the majority of Spry1 is in membrane fraction in MDA-MB-231 cells cultured in growth medium (Fig. 4E), in agreement with the notion of membrane anchorage of Sprys in response to growth factor stimulation^26^. We performed a qPCR array to quantify expression of other genes that characterize either an epithelial or mesenchymal phenotype. Knocking down Spry1 increased E-cadherin and decreased multiple mesenchymal markers including Snail family proteins, the EMT inducers (Supplementary Fig. S6). The down-regulation of Slug was verified by immunoblotting analyses (Fig. 4F).

Since the inhibitory effects of knocking down Spry1 on MDA-MB-231 cell growth and migration are associated with increased E-cadherin *in vitro*, it is important to know whether S1kd tumors have elevated E-cadherin. Immunohistochemistry analyses showed patchy E-cadherin staining both in NT and S1kd tumors, however S1kd tumors had a stronger signal than NT tumors (Fig. 4G). We also performed immunoblotting using tumor extracts and found that S1kd tumors had a trend towards increased E-cadherin expression (Fig. 4H,I). Together, these results indicate that suppression of Spry1 reduces MDA-MB-231 TNBC tumor growth and metastasis, and this effect is associated with an increased epithelial phenotype.

EMT positively correlates with cancer stem-like cell formation. Conversely, a mesenchymal to epithelial transition would be expected to decrease cancer stem cells. Mammosphere assays are commonly used to test the stem cell-like activity of normal and malignant breast cell lines. Thus, we tested mammosphere formation of NT and S1kd MDA-MB-231 cells and found that knockdown of Spry1 decreased MDA-MB-231 cell mammosphere formation (Fig. 4J), and spheres that did form were smaller than NT cells (Fig. 4J–L). These results indicate that endogenous Spry1 functions to maintain the mesenchymal phenotype of MDA-MB-231 cells.

### Knockdown of Spry1 in MDA-MB-231 cells results in activated EGFR degradation and loss of EGF signaling

**Figure 5:**
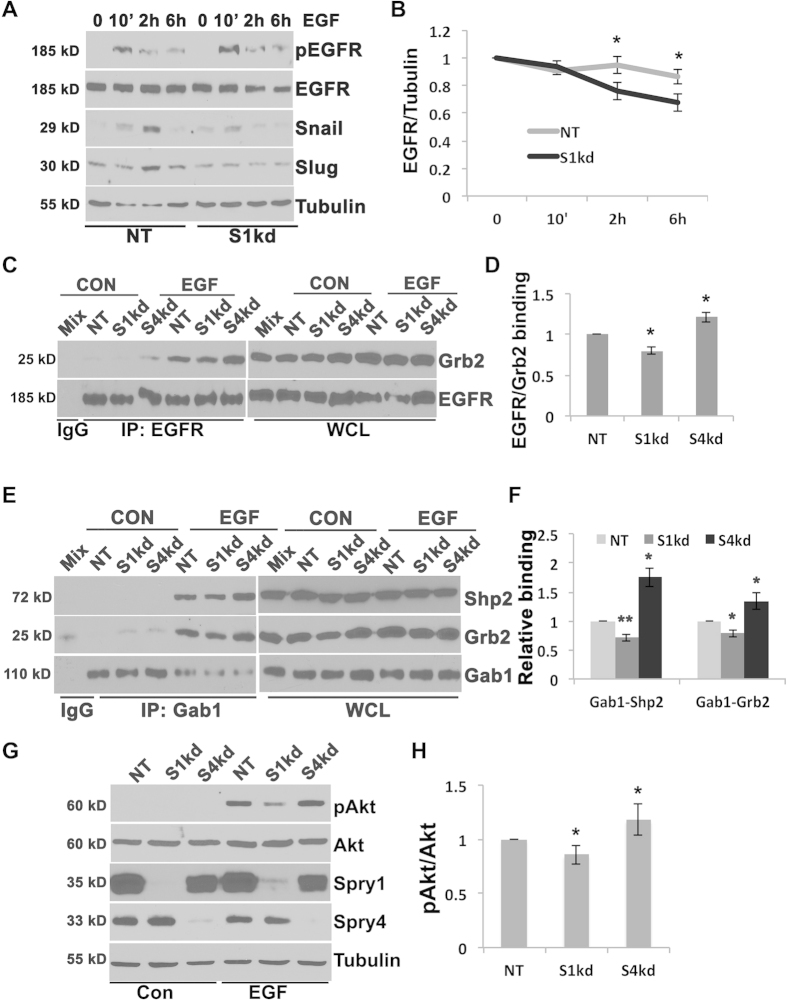
Suppressing Spry1 results in degradation of EGF activated EGFR, and decrease of EGF induced EGFR/Grb2/Gab1/Shp2 cascade complexes formation and downstream signaling, and Snail and Slug induction. (**A**) Time course analysis of Snail, Slug induction, and EGFR protein level upon EGF stimulation. Immunoblotting shows that EGF stimulation induced Snail and Slug expression in NT cells but not in S1kd cells. In NT cells EGF did not change EGFR levels within 6 h, but in S1kd and S4kd cells EGF induced a decrease of EGFR at 2 h and 6 h. The results are representative from at least three independent experiments. The blots were first used for probing pEGFR, Slug or Snail, and then stripped off for reprobing EGFR or Tubulin. (**B**) Quantification to show the trend of EGFR degradation in S1kd cells. (**C**) Immunoprecipitation of EGFR and blotting with Grb2 and EGFR shows decreased EGFR/Grb2 complex formation in S1kd cells compared to NT and S4kd cells. Lysates were incubated with rabbit anti-EGFR overnight, then protein A/G for 1 hr. Beads bound proteins were washed and separated on 8% SDS-PAGE. The same blot was cut between 37 kD and 50 kD markers, and separately probed for EGFR (~185 kD) or Grb2 (~25 kD). (**D**) Quantification of EGF induced EGFR/Grb2 complex formation from three independent experiments. (**E**) Immunoprecipitation of Gab1 and blotting with Grb2, Shp2 and Gab1 shows decreased Gab/Grb2 and Gab1/Shp2 complexes formation in S1kd cells compared to NT and S4kd cells. Lysates were incubated with rabbit anti-Gab1 overnight, then protein A/G for 1 hr. Beads bound proteins were washed and separated on 8% SDS-PAGE. The blot was cut between 37 kD and 50 kD, and between 75 kD and 100 kD, and probed for Grb2 (~25 kD), Shp2 (~72 kD) or Gab1 (~110 kD). (**F**) Quantification of EGFR/Grb2, Gab1/Grb2, Gab1/Shp2 complexes from three independent experiments. (**G**) Representative immunoblotting assay shows decreased EGF mediated pAkt level in S1kd cells compared to NT and S4kd cells. (**H**) Quantification of pAkt/Akt from at least three independent experiments. *p < 0.05, **p < 0.01 relative to controls; white *heavy chain.

Although TNBC lack estrogen and progesterone receptors and do not have HER2 amplification, more than 50% of TNBC express elevated EGFR^27^. EGF/EGFR signaling induces EMT and cancer cell metastasis partly through up-regulation of the Snail family of transcriptional repressors^28^. MDA-MB-231 cells express moderate EGFR, we therefore investigated whether Spry1 knockdown interferes with EGFR signaling and activation of down-stream effectors. Time course analyses showed that EGF stimulation increased Snail and Slug expression in NT control cells, whereas knockdown of Spry1 abolished these effects (Fig. 5A). Spry2 has been shown to stabilize EGFR and potentiate EGFR signaling by binding to c-Cbl and sequestering c-Cbl from interacting with EGFR, thus preventing its degradation^29^,^30^. We tested whether Spry1 modifies Snail and Slug expression through stabilizing EGFR in MDA-MB-231 TNBC cells. Interestingly, unlike in other cell types, we observed no significant EGF mediated EGFR degradation in either NT control (Fig. 5A,B) or parental MDA-MB-231 cells (data not shown). However, knockdown of Spry1 significantly decreased EGFR protein levels at 2 h and 6 h after EGF stimulation (Fig. 5A,B), suggesting that Spry1 likely, at least partly, stabilizes EGFR in MDA-MB-231 cells promotes EGF/EGFR signaling, and Snail and Slug expression. Due to the constitutive membrane anchorage of Spry1 (Fig. 4E), it is also possible that Spry1 somehow interferes EGF mediated EGFR activation. Therefore, we performed immunoprecipitation to examine the status of EGF mediated signaling complex formation. Because MDA-MB-231 cells express both Spry1 and Spry4, and cells with suppression of Spry4 have opposite phenotype with increased proliferation and migration compared to cells with suppression of Spry1 (data not shown), we included Spry4 knockdown cells (S4kd) in our analyses to better elucidate the unique function and underlying mechanism of action of Spry1 in these cells. The results show that EGF stimulation for 10 minutes induced EGFR/Grb2, Grb2/Gab1 and Gab1/Shp2 complex formation in NT control cells, while knocking down of Spry1 decreased the formation of these complexes (Fig. 5C–F). Conversely, knockdown of Spry4 increased EGFR down-stream signaling complex formation (Fig. 5C–F). As a consequence, knockdown of Spry1 attenuated, whereas knockdown of Spry4 potentiated EGF mediated Akt phosphorylation (Fig. 5G,H). Together, these results suggest that Spry1 uniquely promotes MDA-MB-231 TNBC cell mesenchymal phenotype likely through stabilizing EGFR and/or facilitating EGFR activation and the formation of down-stream signaling complexes.

### Spry1 functions similarly in multiple TNBC cells

**Figure 6:**
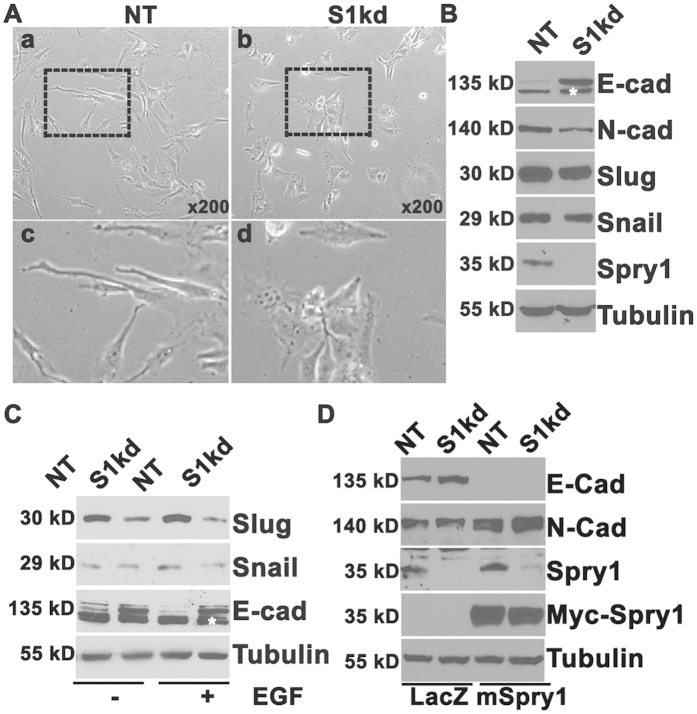
Knockdown of Spry1 in MDA-MB-157 TNBC has similar effect in inducing an epithelial phenotype, and re-expression of Spry1 reverses this effect. (**A**) Representative phase contrast images show a fibroblastic-like phenotype in NT cells and an epithelioid phenotype in S1kd cells. (**B**) Immunoblotting assay shows increased E-cadherin but decreased N-cadherin, Slug and Snail expression in S1kd cells compared to NT cells. (**C**) Immunoblotting assay shows an induction of Snail and Slug proteins by EGF stimulation at 3 h in NT but not in S1kd cells. (**D**) Re-expression of mSpry1 in S1kd MDA-MB-157 cells restored N-cadherin expression, and inhibited E-cadherin expression. All blots were reused for probing Tubulin. The results are representative of at least three independent experiments. White *non-specific band.

To test whether the effect of Spry1 is limited to MDA-MB-231 cells, we knocked down Spry1 using shRNA in the MDA-MB-157 cell, another basal-like TNBC cell line that has somewhat lower level of Spry1 expression compared to MDA-MB-231 cells. Figure 6A shows that knockdown of Spry1 significantly changed MDA-MB-157 cell morphology from fibroblast-like to a polygonal epithelial-like morphology. Knockdown of Spry1 also significantly increased E-cadherin, and decreased N-cadherin, Snail and Slug protein levels under normal conditions in growth medium (Fig. 6B). In addition, EGF mediated up-regulation of Snail was also abolished in S1kd MDA-MB-157 cells (Fig. 6C). Next, we re-expressed myc tagged mouse Spry1 into S1kd MDA-MB-157 cells to determine if these effects were caused specifically by loss of Spry1. Knockdown of Spry1 was verified with mouse monoclonal antibody specifically against human Spry1 (Santa Cruz Biotechnology, sc-365520), and re-expression of mSpry1 was verified with anti-Myc antibody. Indeed, introducing mSpry1 restored N-cadherin expression and decreased E-cadherin expression of S1kd MDA-MB-157 cells (Fig. 6D). Furthermore, we also observed an epithelial like morphology in Spry1 knockdown Hs578T (HTB-126 cells), a cell line that was maintained in medium containing 10 μg/ml insulin (Supplementary Fig. S7). Suppressing Spry1 in Hs578T cells inhibited cell migration measured by scratch assay, and decreased the subpopulation that expressing CD61 (β3-integrin), a cell surface molecular involved in cell adhesion and migration (Supplementary Fig. 7S). These data again support the idea that endogenous Spry1 in at least a subset of TNBC contributes to the mesenchymal phenotype, thus promoting malignancy. Our results suggest that suppressing Spry1 in these TNBC will suppress tumorigenicity by causing a reversion to epithelial morphology.

## Discussion

Our study shows that Spry1 is a novel target that may play a specific role in the malignancy of at least a subset of human TNBC. Endogenous Spry1 constitutively anchors to the cell membrane, stabilizes EGFR protein, or facilitates EGF mediated EGFR activation and EGFR/Grb2/Gab1/Shp2 cascade complexes formation and signaling. This signaling maintains the malignant phenotype, including characteristics of mesenchymal morphology, high rates of cell growth and migration, and clonal growth ability. Suppressing Spry1 reverses these phenotypes by impairment of EGFR signaling, and leads to a significant reduction in tumor growth *in vivo.*

Spry proteins function as tumor suppressors in multiple cancers including non-classified breast cancer, lung and prostate cancers, where expression of Spry genes is down-regulated^18^,^19^,^20^,^31^. On the other hand, expression of Sprys has also been reported to facilitate Ras or Raf mediated cell transformation and tumorigenesis^21^,^24^. In addition, in colon cancer, Spry2 functions as an oncogene via up-regulation of c-Met expression^22^. Therefore, the expression of Spry proteins and their roles in cancers are complex and cell context-dependent. Sprys are induced by MAPK activation and act as feedback regulators of RTK/MAPK signaling pathway in many types of normal cells. Up-regulation of Spry proteins in cells with Ras or Raf activating mutations or inappropriate activation of RTKs is not unexpected. Thus, the level of expression of Sprys may indicate an abnormal activation of RTK/Ras/Raf/MAPK signaling. MDA-MB-231 cells express an active mutant of Ras^32^. MEK inhibitor U0126 treatment significantly decreases Spry1 and Spry4 protein levels in MDA-MB-231 cells (Supplementary Fig. 8S), suggest that the constitutive activation of MAPK/ERK signaling pathway is involved in the up-regulation of Spry1 and Spry4 in these cells. TNBC is a subtype of aggressive breast cancer with different gene mutations, and more than 50% of TNBC have high expression of EGFR^27^, whose signaling may also induce Spry proteins expression through canonic stimulation of the ERK pathway. In a meta-analysis of the gene expression profiles of a total of 1,107 tumors, Faratian *et al.* has reported that *Spry1*, *Spry2* and *Spry4* differentially expressed across clinicopathological subgroups of the breast cancer^33^. Owing to the high diversity of TNBC in terms of gene expression profiles and histomorphology^34^,^35^, our initial result of moderate to high Spry1 expression in a small non-classified TNBC cohort suggests that the expression of Spry1 may also be TNBC subtype and/or pathology stage dependent. Further study is warranted to clarify whether Spry1 is an indicator of a subtype of TNBC and/or a pathological stage with abnormal MAPK pathway activation. The mechanism in regulation of Spry family members is diversity. Promoter hypermethylation has been shown to contribute to the down-regulation of Spry2 in prostate cancer^36^. However, the decreased Spry1 expression in prostate cancer mainly attributes to other mechanisms of gene inactivation such as alterations in transcriptional factors and microRNA mediated post-transcriptional gene silencing^37^. Our study indicates there are different mechanisms in regulation of Spry family expression in TNBC.

The precise mechanism by which Spry proteins regulate RTK signaling pathways remains unclear because Spry proteins bind many components of the RTK/ERK pathway, including Grb2, Shp2, Sos, and Raf1, as well as other signaling molecules, such as c-Cbl, TESK and CIN85^38^,^39^. Spry proteins also act at the level of RTK and regulate ligands induced RTK turn over to ensure appropriate cellular signaling. Spry2 can stabilize EGFR by binding and sequestering c-Cbl, which mediates EGFR degradation, and suppression of Spry2 impairs EGF mediated EGFR signaling^30^. We have previously shown Spry1 stabilizes FGFR in chondrocytes in regulating chondrogenesis^40^. In this study, we demonstrate that MDA-MB-231 cells have high level of Spry1 coincident with impaired process of EGF induced EGFR turn over that may contribute, at least partly to their malignancy. The tyrosine phosphorylation of Spry2 induced by EGF/EGFR signaling is required for its membrane translocation and c-Cbl binding in stabilizing EGFR^30^. However, we observed a constitutive membrane localization of Spry1 in MDA-MB-231, and were unable to detect Spry1 tyrosine phosphorylation (data not shown). It has been shown that palmitoylation of Sprys’ cysteine-rich domain is associated with their membrane localization in FGF2 stimulated or exponentially growing endothelial cells^26^. The optimal growth condition *in vitro* as well as the active Ras mutation in MDA-MB-231 cells may contribute to the constitutive membrane anchorage of Spry1 in these cells. In support, we observed an increased cytoplasmic expression of Spry1 in growth medium cultured MDA-MB-231 cells when treated with either MEK or PI3K inhibitor, or withdrawal of serum for 24 hrs (Supplemental Fig. 9S). Therefore, the membrane translocation of Spry1 from cytoplasmic is likely induced by activation of growth factor receptor(s) such as EGFR, and then maintains or facilitates their activation in MDA-MB-231 cells. It is known that solid tumor cells are in a relative hypoxic environment, and this less optimal growth condition may be associated with the major cytoplasmic cellular localization of Spry1 observed by tumor sample immunohistochemistry. Alternatively, the functional activity and localization of Spry1 may be associated with the disease pathological stages with aberrant activation of growth factor signaling pathways. Further detailed study on a larger sample size is needed to clarify this discrepancy.

The EGF induced EGFR signaling involves EGFR auto-phosphorylation, and effective EGFR/Grb2/Gab1/Shp2 cascades complex formation. Knockdown of Spry1 impaired this complex formation, it is also possible that the membrane localized Spry1 functions as scaffold protein to interfere EGFR auto-phosphorylation and/or EGFR/Grb2/Gab1/Shp2 complex formation, further detailed analysis is needed to address the mechanism.

**Figure 7:**
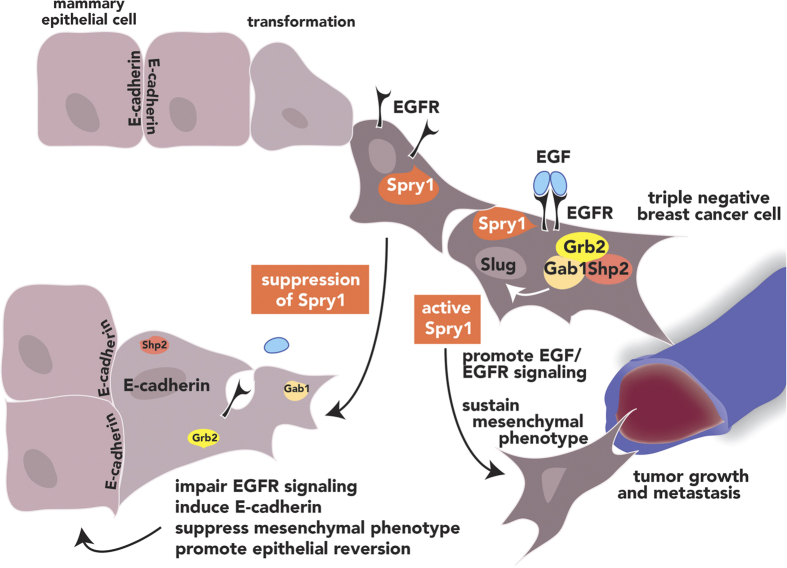
Summary of Spry1 activity in regulating TNBC cell malignancy. Spry1 stabilizes EGFR protein and its down-stream signaling, which promotes the mesenchymal phenotype and malignancy of TNBC. Loss of Spry1 leads to the degradation of EGF activated EGFR, and impairs EGFR signaling and cancer cell malignancy.

EGFR signaling has been shown to induce EMT through various mechanisms including endocytosis of E-cadherin, as well as Snail or Twist expression, which leads to a reduction in E-cadherin^16^,^41^,^42^. Epithelial-mesenchymal transition plays important role in cancer cell metastasis and invasion, as well as cancer stem cell development^43^. Loss of membranous E-cadherin and positive mesenchymal markers expression are significantly associated with poor clinical outcome of TNBCs^44^,^45^,^46^. Although we were unable to show whether there is an inverse correlation between membranous E-cadherin and Spry1 expression due to lack of available antibodies for co-immunostaining, we observed a variable increased expression of Fibronectin, a mesenchymal marker involved in tumor cell migration and metastasis^47^, concomitant with the high expression of Spry1 in some of TNBC (Supplemental Fig. S10). Further study by RT-qPCR and immunoblotting analyses on a larger sample size is needed to clarify whether there is an inverse correlation of Spry1 expression and mesenchymal phenotype in TNBC tissues. In conclusion, our study identifies a novel mechanism where Spry1 stabilizes EGFR and/or facilitates EGFR signaling, promotes EGF mediated Snail and Slug expression, and sustains at least a subset of TNBC mesenchymal phenotype that is described in Fig. 7. These characteristics suggest that suppressing Spry1 in these TNBC would provide therapeutic value by promoting the differentiated phenotype of this subtype of mammary epithelial cell carcinoma.

## Methods

### Human breast cancer tissue microarray immunohistochemistry assay

Paraffin embedded surgical resection specimens of 17 TNBC, 6 non-TNBC and their available corresponding normal breast tissues were obtained from the BioBank at Maine Medical Center Research Institute. Tissue microarrays were generated by the Histopathology Core at Maine Medical Center Research Institute. Estrogen and progesterone receptor expression was verified by immunohistochemistry. Spry proteins were detected by immunostaining using Spry1, Spry2 and Spry4 antibodies (Santa Cruz), followed by incubation of SignalStain Boost IHC Detection Reagent (HRP, rabbit, Cell Signaling Technology) and DAB peroxidase substrate (Vector Laboratory). Procedures using human tissue were approved by the Institutional Review Board at Maine Medical Center and conducted in compliance with ethical and safe research practices involving human subjects or tissues.

### Cell culture

MCF10A, MCF7, MDA-MB-231, MDA-MB-157, Hs578T (HTB-126) and MDA-MB-468 cell lines (ATCC) were cultured in the following media. MCF10A: α MEM/F-12 (50/50) containing 5% horse serum supplemented with 100 ng/ml cholera toxin (Sigma), 10 μg/ml insulin (Sigma), 20 ng/ml EGF(R&D) and 1x antibiotic-antimycotic mixture (Atlanta Biologicals, Lawrenceville, GA. USA); MCF7: αMEM containing 10% FBS and 10 μg/ml insulin and 1x antibiotic-antimycotic mixture; MDA-MB-231: αMEM containing 10% FBS supplemented, 1% non-essential amino acids (Invitrogen) and 1x antibiotic-antimycotic mixture. MDA-MB-157: L-15 containing 10% FBS with 1x antibiotic-antimycotic mixture; Hs578T: DMEM containing 10% FBS and 10 μg/ml insulin with 1x antibiotic-antimycotic mixture; MDA-MB-468: L-15 containing 10% FBS with 1x antibiotic-antimycotic mixture. All cells were cultured in a humidified atmosphere containing 5% CO_2_ except MDA-MB-468 in a free gas exchange with atmospheric air. To generate stable Spry1 knockdown cells, MDA-MB-231 cells (passage 6 to 9), or MDA-MB-157 were transduced with human Spry1 shRNA or non-targeting control lentiviruses (Open Biosystems), and selected in medium containing 2 μg/ml puromycin.

### Western blotting and immunoprecipitation

Cells were lysed in HNTG buffer (50 mM HEPES, 150 mM NaCl, 10% glycerol, 1% Triton X-100, 1.5 mM MgCl_2_, 1 mM EGTA, 1 mM NaF, 1 mM NaVO_3_ and proteinase inhibitor cocktail (Roche)). Cell lysates were subjected to SDS-PAGE and proteins were transferred onto nitrocellulose membranes. Immunoblotting was performed with antibodies recognizing Spry1, Spry2, Spry4, ERK, E-cadherin (CDH) (Santa Cruz Technology), phospho-ERK, phospho-Akt, Akt, EGFR, Snail and Slug (Cell Signaling Technology) and tubulin (Sigma). For immunoprecipitation, lysates were incubated with rabbit antibodies against EGFR, Gab1, c-Cbl (Cell Signaling Technology), Grb2, Shp2 (BD Bioscience) or Spry1 (New England Peptide LLC , rabbit polyclonal antibody produced by immunizing animals with a synthetic peptide corresponding to human Spry1 aa143–156, the sequence of the peptide is Ac-CRPVPGHRSERAIRT-amide), then precipitated with protein A/G plus agarose beads (Santa Cruz Technology). Beads were washed three times and boiled for immunoblotting analysis using mouse monoclonal antibodies against Grb2, Shp2, Gab1 (BD, Bioscience), EGFR (Cell Signaling Technology), and Spry1 (Santa Cruz). Immunoblots were quantified using ImageJ (NIH).

### Cell growth analysis and colony forming assay

For growth curves, Spry1 knockdown (S1kd) and non-targeting control (NT) stable MDA-MB-231 cells were plated at 5 × 10^3^ cells/well in 12 well plates in triplicate, and media replaced every two days. Cells were counted every two days using a Coulter counter. For measurement of anchorage-independent colony formation, 5 × 10^3^ cells were mixed with medium containing 0.4% agar and were spread on top of a bottom agar layer (0.8% agar). Cells were grown for 3 weeks. Colonies were fixed and stained with crystal violet, and counted and measured by using ImageJ (NIH).

### Cell cycle analysis and EdU incorporation

NT or S1kd MDA-MB-231 cells were cultured in complete media or serum-free media for 24 h. Cells were trypsinized and washed with cold PBS, fixed in 70% ethanol, stained with propidium iodide and analyzed on FACSCalibur (BD). For EdU incorporation, cells were labeled with 5′-ethynyl-2′-deoxyuridine for 5 h and stained with a Click-iT EdU Alexa Fluor 488 Imaging kit according to the manufacturer’s instructions (Invitrogen).

### Migration assay

NT or S1kd MDA-MB-231 cells were plated in 6 well plates in triplicates at subconfluence and cultured for 24 h. Confluent cells were treated with 2 μg/ml mitomycin C for 2 h prior to making a scratch denudation with a 1 ml pipette tip. Cells were washed with growth medium and continually cultured in growth medium containing 1 μg/ml mitomycin C for 48 h. Cell migration was photographed in eight regions at 0, 24 and 48 h under a phase microscope. Denuded areas were measured and calculated with ImageJ.

### Invasion assay

The invasion assay was performed with Matrigel-coated transwell membrane filter inserts in 24-well culture plates (BD Biosciences). NT and S1kd MDA-MB-231 cells were trypsinized, counted, and added to the upper chambers of transwell inserts with 8 μm pores. A total of 1 × 10^4^ cells in a volume of 200 μl was added into each insert, and 600 μl of αMEM containing 10% FBS was added to the lower chamber. Cells were incubated at 37 °C for 24 h. Cells that remained in the inserts were removed, and cells that migrated to the underside of the inserts were fixed and stained with DAPI. The migrated cells were photographed under a fluorescence microscope and counted from five randomly selected fields. The experiment was performed in triplicate.

### Mammosphere assay

Mammosphere assays were performed as described by Dontu^48^ with modifications. Briefly, NT or S1kd MDA-MB-231 cells were trypsinized and counted. 5000 cells were suspended in serum-free αMEM containing 20 ng/ml FGF2, 20 ng/ml EGF (R&D Systems) and 1xB27 serum free supplement (Invitrogen) and cultured on ultra-low attachment 6-well plates. Mammoshperes were monitored daily by phase-contrast microscopy to ensure that they were derived from single cell. The number of mammospheres was counted at day 10, and images were obtained under microscope using cannon EOS camera. Mammosphere size was measured using ImageJ (NIH).

### Mouse xenografts

Six to eight-week old NOD/SCID female mice (Jackson Laboratory) were used for xenograft tumor studies. NT and S1kd MDA-MB-231 stable cell lines were harvested in the exponential growth phase using EDTA solution and washed twice with ice cold PBS, and resuspended in PBS at concentrations of 1 × 10^6^, 1.5 × 10^6^ and 2 × 10^6^ per 200 μl. 200 μl of cells were injected into the left inguinal mammary fat pad, and four mice were injected per group. Tumor size was measured with calipers weekly and volume calculated using the formula W^2^L/2 (L = length, W = width)^49^. Six to nine weeks later when tumors were approximately 10–15 mm in length, tumors and lungs were removed and snap frozen or fixed in 10% formalin for further analysis. All procedures involving animals were conducted in compliance with a protocol approved by the Institutional Animal Care and Use Committee of Maine Medical Center.

### RT-qPCR

Total RNAs were extracted using the Qiagen RNeasy mini kit. The concentration was measured with a NanoDrop Spectrophotometer (NanoDrop Technologies) at 260 nm/280 nm. The ratios of 260 nm/280 nm readings of all samples were between 1.8 and 2.0. ProtoScript M-MuLA First Strand cDNA Synthesis kit (Biolab) was used to generate cDNA. Quantitative real-time PCR (qPCR) of target genes was performed using SYBR Green (SABiosciences) on an IQ5 Multicolor Real-Time PCR Detection System (BioRad) according to the manufacturer’s instructions. GAPDH was used as an internal reference in each reaction. Melting curve analyses using the program run in the step acquisition mode was used to verify the presence of a single amplification production. Primers for qPCR are shown in supplementary Table S1. EMT qPCR array was performed using Qiagen human EMT PCR array kit according to the manufacturer’s instructions.

### Statistical Analysis

Results are presented as means + S.D. and analyzed with two-way ANOVA and two-tailed *t*-tests. *P* < 0.05 was considered as statistically significant.

## Additional Information

**How to cite this article**: He, Q. *et al.* Suppression of Spry1 inhibits triple-negative breast cancer malignancy by decreasing EGF/EGFR mediated mesenchymal phenotype. *Sci. Rep.* **6**, 23216; doi: 10.1038/srep23216 (2016).

## Supplementary Material

Supplementary Information
