# Corrigendum: Suppression of Spry1 inhibits triple-negative breast cancer malignancy by decreasing EGF/EGFR mediated mesenchymal phenotype

**DOI:** 10.1038/srep46791

**Published:** 2017-05-02

**Authors:** Qing He, Hongyu Jing, Lucy Liaw, Lindsey Gower, Calvin Vary, Shucheng Hua, Xuehui Yang

Scientific Reports 6: Article number: 2321610.1038/srep23216; published online: 03 15 2016; updated: 05 02 2017

The Acknowledgements section in this Article is incomplete.

“We thank Dr. Robert Friesel for critical reading of the manuscript. We thank MMCRI histology core Armie Mangoba, Katrina Abramo and Dr. Volkhard Lindner for creating breast cancer tissue array (TMA) and histochemistry analysis. We thank MMC BioBank Robert Ackroyd, Dr. Ivette F. Emery and Dr. Anne Breggia for coordinating studies on clinical specimens. This study was supported by the Maine Cancer Foundation Accelerate Grant and a Maine Medical Center Research Program Grant to X.Y”.

Should read:

“We thank Dr. Robert Friesel for critical reading of the manuscript. We thank MMCRI histology core Armie Mangoba, Katrina Abramo and Dr. Volkhard Lindner for creating breast cancer tissue array (TMA) and histochemistry analysis. We thank MMC BioBank Robert Ackroyd, Dr. Ivette F. Emery and Dr. Anne Breggia for coordinating studies on clinical specimens. This study was supported by the Maine Cancer Foundation Accelerate Grant and a Maine Medical Center Research Program Grant to X.Y. This work was supported by a grant from the Maine Cancer Foundation and NIH grants P30 GM1033465 (molecular phenotyping, histopathology, and progenitor cell analysis cores) to D. Wojchowski, PI, and P30 GM103392 (Histopathology and cell imaging cores) to R. Friesel, PI, and generous support from the Maine Medical Center”.

