# Supplementary material for: Suppression of Spry1 inhibits triple-negative breast cancer malignancy by decreasing EGF/EGFR mediated mesenchymal phenotype
Source: Sci Rep. 2016 Mar 15;6:23216. doi: 10.1038/srep23216 (PMC4791662; doi:10.1038/srep23216)
Supplement: Supplementary Information [file srep23216-s1.pdf]

Title: Suppression of Spry1 inhibits triple-negative breast cancer malignancy by decreasing EGF/EGFR mediated mesenchymal phenotype

Qing He[1,2], Hongyu Jin[1,3], Lucy Liaw[1], Lindsey Gower[1], Calvin Vary[1], Shu-chen Hua[3], Xuehui Yang*[1]

Suppplementary Table S1. Primers for RT-qPCR

Gene name	Primer sequences	Gene bank number
E-cadherin	Sense: 5'CTGGGACTCCACCTACAGAAAGTT -3'<br>Antisense: 5'-CAGAAACGGAGGCCTGAT-3'	NM_004360.3
N-cadherin	Sense: 5'-AGCCAAGCACTGTCAGGA-3'<br>Antisense: 5'-ACAATGGATGGGAAAACAG-3'	NM_001613.2
Slug	Sense: 5'-GGTGACTTCAGAGGCGCCGG-3'<br>Antisens: 5'-GGCGGTCCCTACAGCATCGC-3'	NM_003068.7
Snail	Sense: 5'-TATGCCGCGCTCTTTCCTCGTC-3'<br>Antisense: 5'-CGGTGGGGTTGAGGATCTCCG-3'	NM_005985.3
GAPDH	Sense: 5'-GAAGGTGAAGGTCGGAGTC-3'<br>Antisens: 5'-GAAGATGGTGATGGGATTTC-3'	NM_002046.3

Supplementary Table S2

Spry member	Localization	Immunostaining intensity		
		Normal breast tissue	TNBC (% of total)	Non-TNBC
Spry1	Basal cells and fibroblasts	moderate	weak (2/17, 12%)<br>moderate (11/17, 65%)<br>strong (4/17, 24%)	weak (4/6, 67%)
Spry2	Ductal epithelium	moderate	weak (7/17, 41%)<br>moderate (6/17, 35%)<br>strong (4/17, 24%)	weak (5/6, 83%)
Spry4	Ductal epithelium	moderate	weak (0/17, 0%)<br>moderate (10/17, 59%)<br>strong (7/17, 41%)	moderate (6/6, 100%)

Supplementary Table S2. The comparison of Spry proteins of TNBC and non-TNBC versus normal breast tissues. Immunohistochemistry (IHC) analysis of Spry proteins on a tissue array consist of 17 TNBC, 6 non-TNBC and available normal breast tissues. The signal intensity of IHC of Spry1, Spry2 and Spry4 in normal breast tissues was scored as moderate, the staining intensity of breast cancers was compared to that in normal breast tissue and scored as weak, moderate or strong accordingly. Down-regulation of Spry1 and Spry2 was observed in majority non-TNBC compared to normal breast tissues (67% and 83% respectively). Whereas in TNBC, the protein levels of Sprys were not significantly decreased compared to normal breast tissues.

Supplementary Figure S1

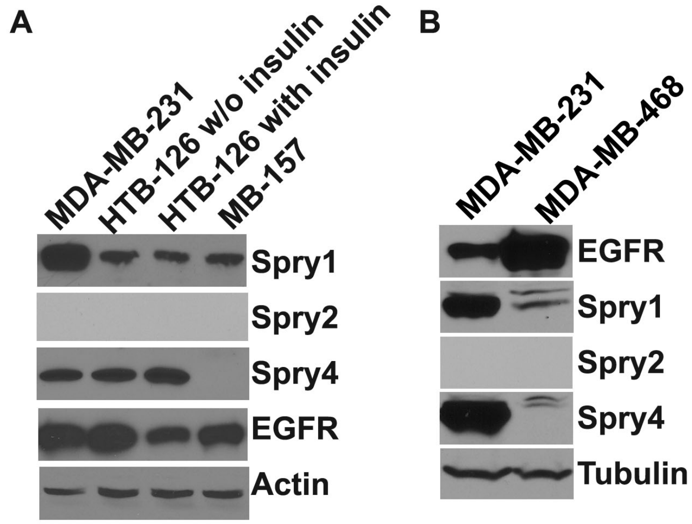

Supplementary Figure S1. Immunoblotting analysis to examine Sprys expression in Hs578T (HTB-126) and MDA-MB-468 TNBC cells. A) HTB-126 cells were growing in DMEM containing 10% FBS and 10μg/ml insulin according to ATCC instruction. To examine if insulin has effects on Sprys expression, we withdrew insulin 24 hours prior to lyse cells. Cell lysates from MDA-MB-231, HTB-126 cells growing with or without (w/o) insulin and MB-157 were separated on 8% SDS-PAGE and immunoblotted using antibodies against Spry1, Spry2, Spry4, EGFR and actin. B) MDA-MB-468 cells were growing in Leibovitz's L-15 containing 10% FBS at 37^0C incubator without CO_2 (****A CO_2 and air mixture is detrimental to cells for cultivation) according to ATCC instruction. MDA-MB-231 and MDA-MB-468 Cell lysates were separated on 8% SDS-PAGE and immunoblotted using antibodies against Spry1, Spry2, Spry4, EGFR and tubulin.

Supplementary Figure S2

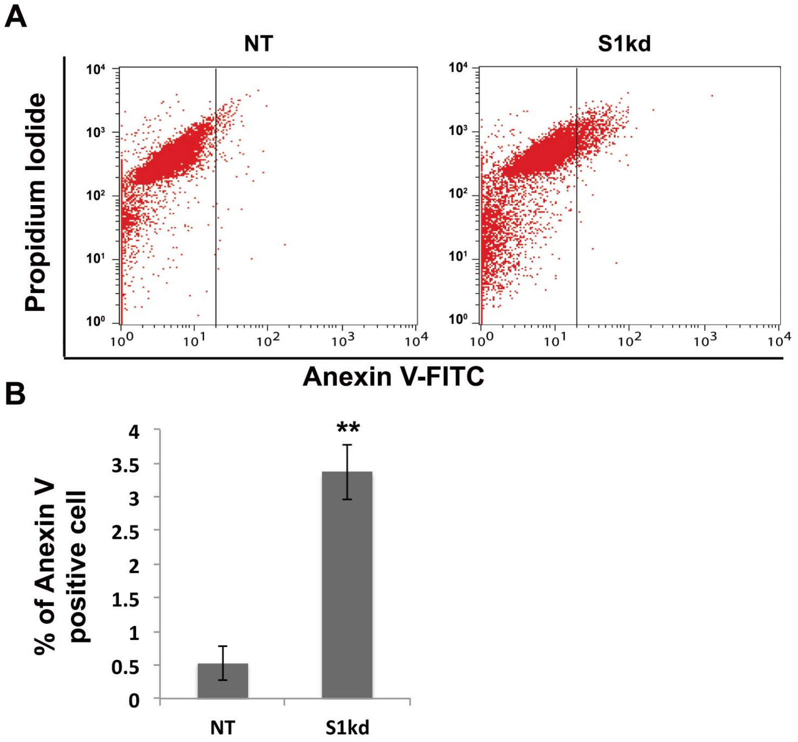

Supplementary Figure S2. Annexin V immunofluorescence staining followed by FACS analysis to examine the cell apoptosis. A) Representative FACS analyses. B) Graphic illustration shows that knocking down Spry1 increased MDA-MB-231 cell apoptosis. **: $p<0.01$.

Supplementary Figure S3

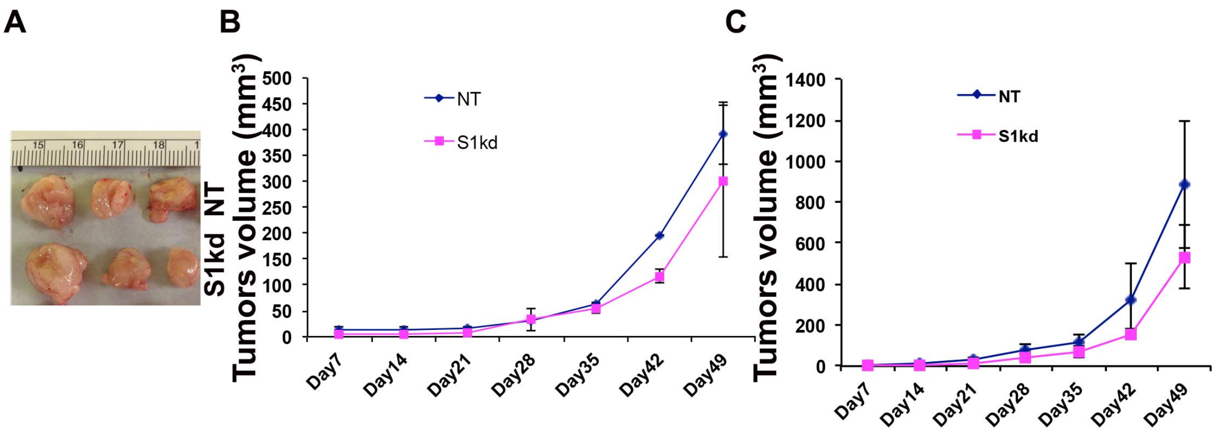

Supplementary Figure S3. Knocking down of Spry1 decreases MDA-MB-231 tumor growth. A) Images of tumors harvested at 7 weeks after fat pad inoculation of $2x10^6$ S1kd or NT MDA-MB-231cells. B) Tumor growth curve from $1.5x10^6$ cell inoculation. C) Tumor growth curve from $2x10^6$ cell inoculation.

Supplementary Figure S4

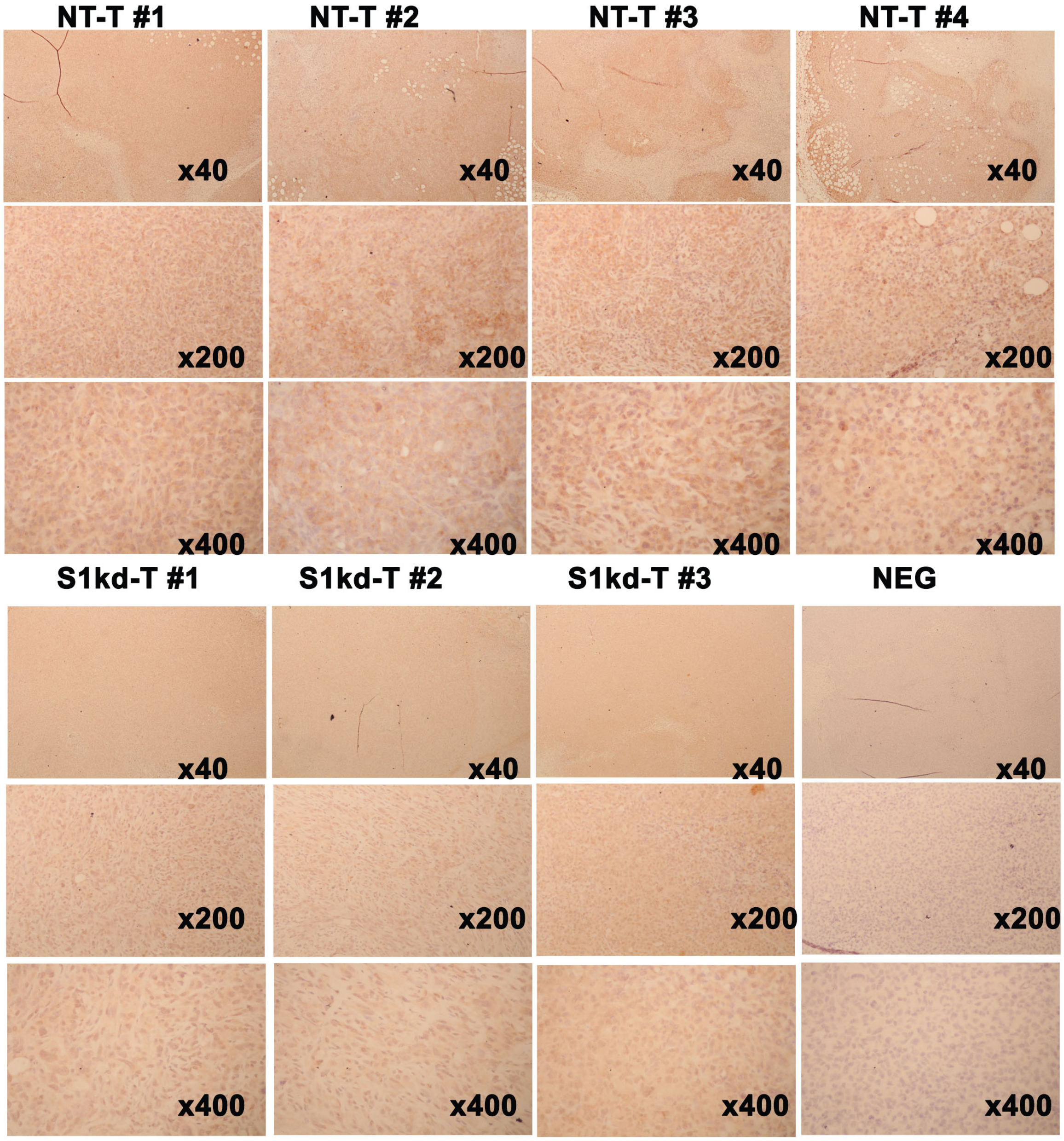

Supplementary Fig. S4. Immunohistochemistry analysis of Spry1 expression in MDA-MB-231 cell xenograft tumors. Tumors were harvested at 9-weeks from 1x10^6 NT or S1kd MDA-MB-231 cells injected mice, fixed and embedded for analysis. Sections were immunostained with anti-Spry1, and represent images were obtained with Cannon EOS. The results show a roughly 50-60% decrease of Spry1 expression in S1kd tumors compared to NT tumors.

Supplementary Figure S5

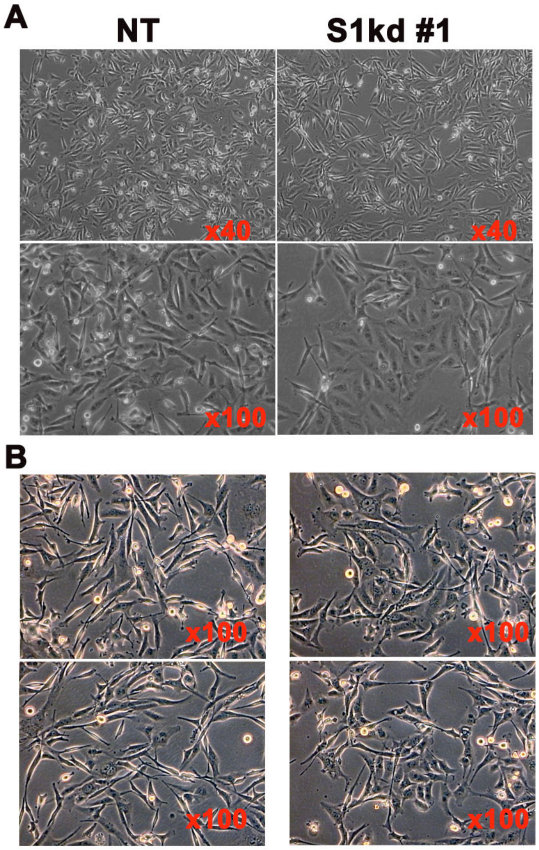

Supplementary Fig. S5. Spry1 knockdown MDA-MB-231 cells form epithelial-like patches. A) Represent phase contrast images from low passage of MDA-MB-231 cells (passage 8, newly received MDA-MB-231 cells from ATCC was referred as passage 0) that were used for knocking down Spry1. B) Represent phase contrast images from high passage of MDA-MB-231 cells (passage 52) that were used for knocking down Spry1.

Supplementary Figure S6

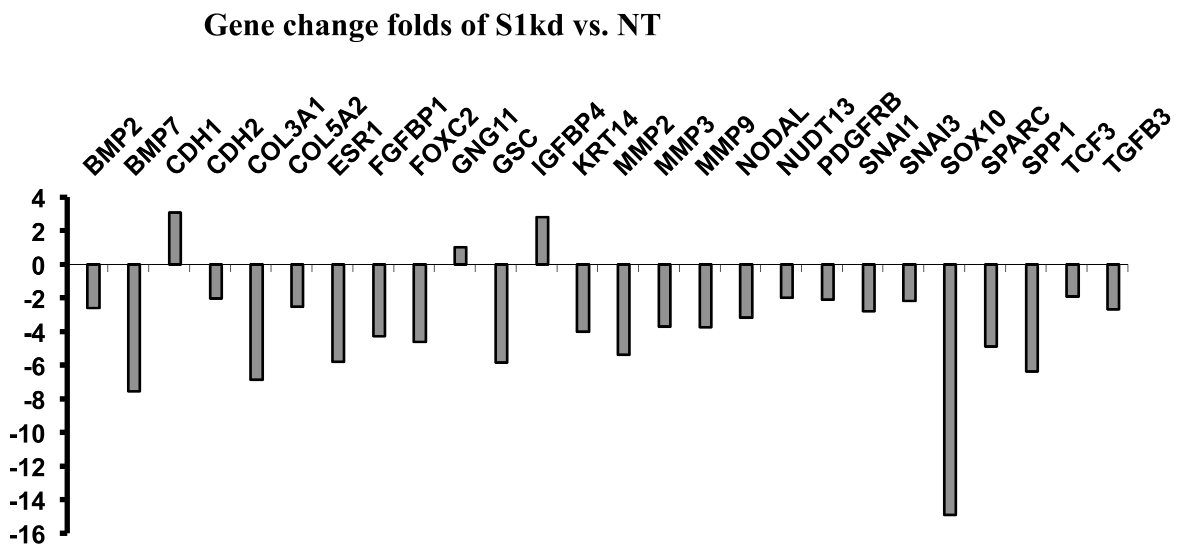

Supplementary Figure S6. RT-qPCR array analysis of multiple epithelial and mesenchymal markers to evaluate the effect of knocking down Spry1 in MDA-MB-231 cells mesenchymal phenotype. Knockdown of Spry1 increased E-cadherin (CDH1) mRNA, decreased N-cadherin (CDH2) and multiple mesenchymal markers such as Snai1, Snail3, FoxC2 compared to NT control.

Supplementary Figure S7

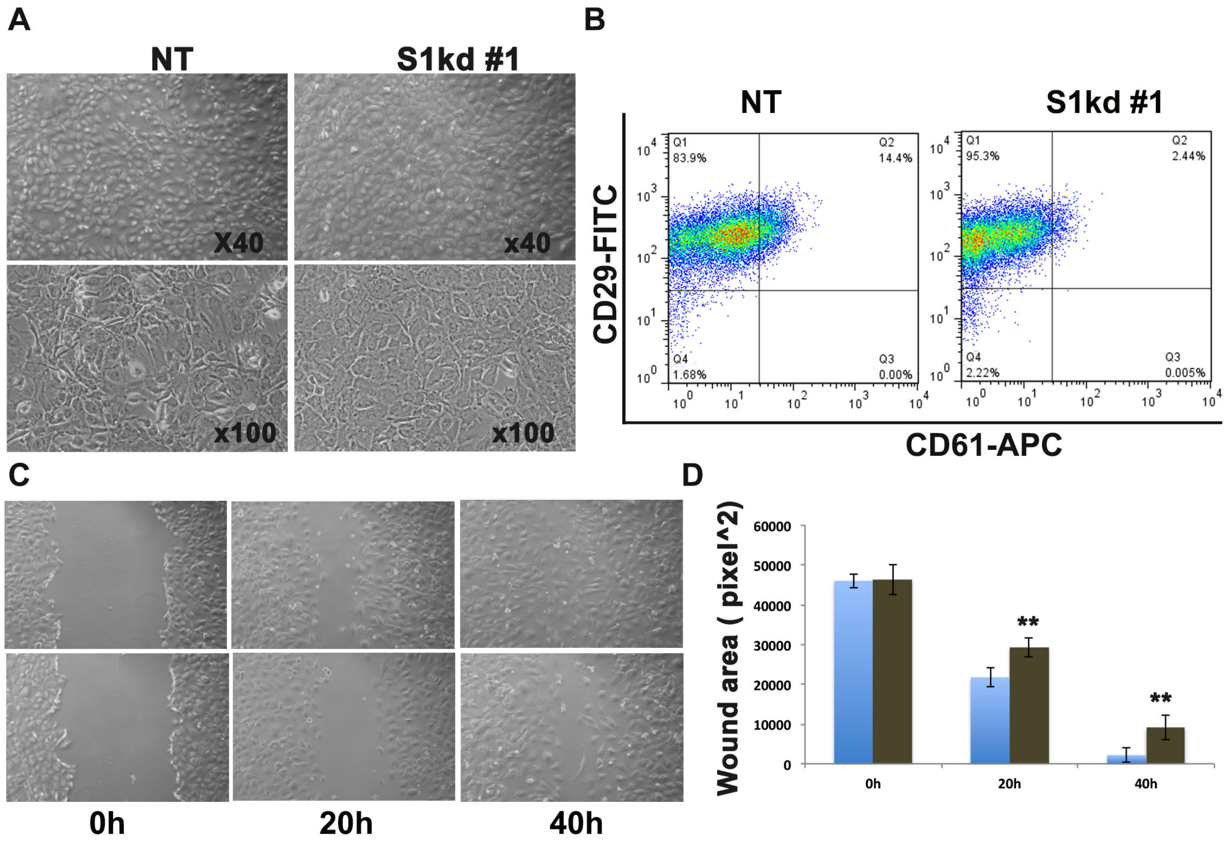

Supplementary Fig. S7. Knockdown of Spry1 in HTB-126 cells results in epithelial like morphology change and decreases cell migration and CD61 (β3-integrin) subpopulation. A) represent phase contrast images from Spry1 knockdown or NT HTB-126 cells. B) FACS analysis of S1kd #1 or NT HTB-126 CD29 (β1-integrin) and CD61 integrin, which are important for cell adhesion and migration. C) Scratch assay shows Spry1 knockdown cells migrated slower than NT cells. D) Quantification of wound areas from scratch assay in C.

Supplementary Figure S8

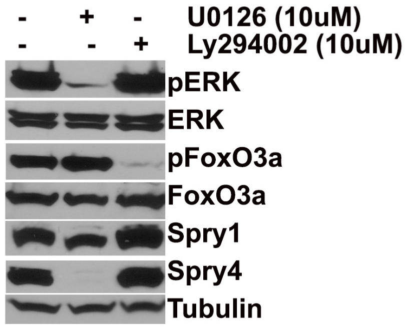

Supplementary Figure S8. Activation of MEK/MAPK signaling pathway contributes to the expression of Spry1 and Spry4 in MDA-MB-231 cells. MDA-MB-231 cells were cultured in αMEM containing 10% FBS and treated with 10μM MEK inhibitor U0126 or PI3K inhibitor Ly294002 for 24h. Cell lysates were subjected for immunoblotting using indicated antibodies. MEK inhibitor U0126 but not PI3K inhibitor Ly294002 treatment significantly reduced Spry1 and Spry4 expression.

Supplementary Figure S9

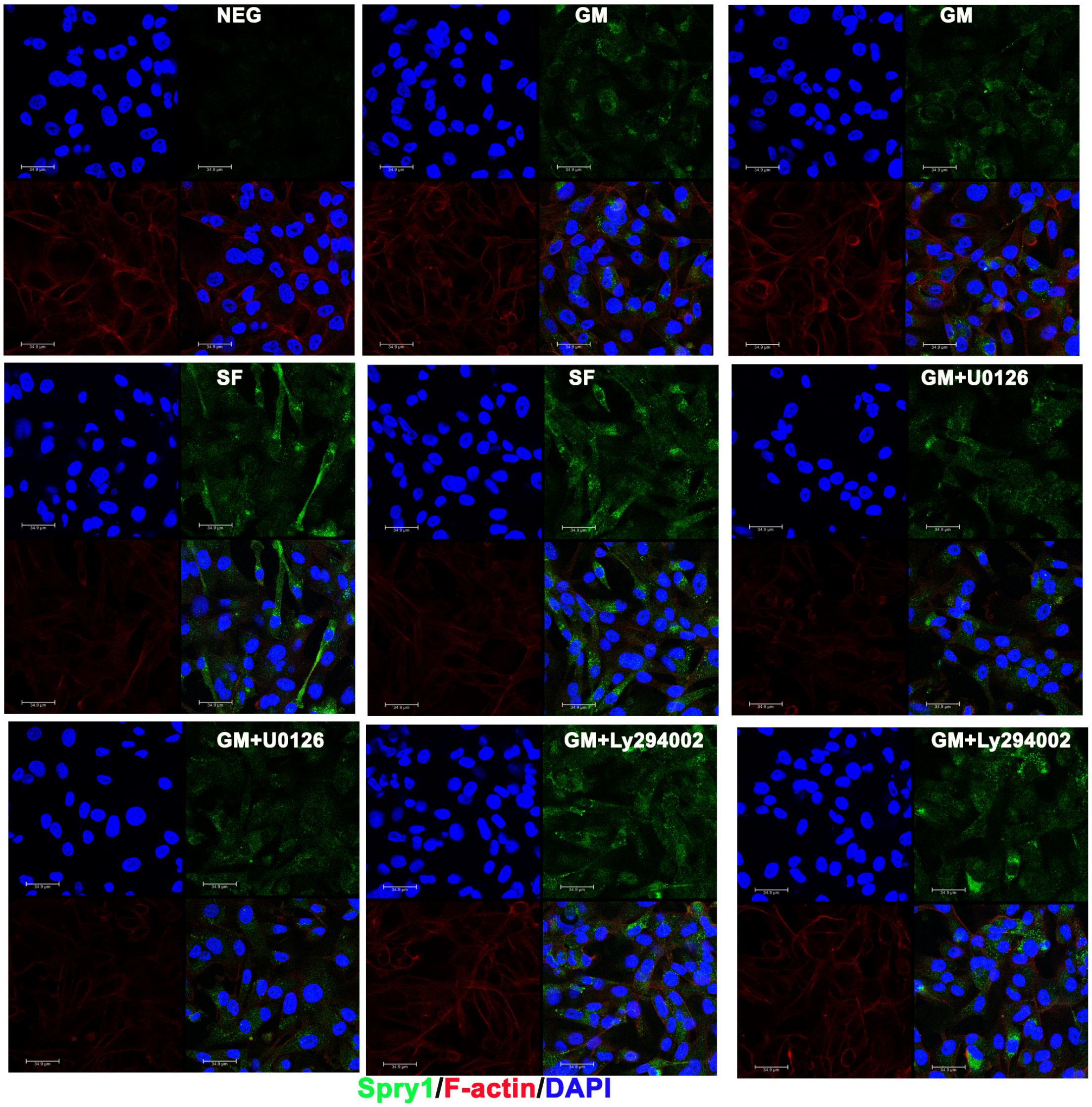

Supplementary Fig. S9. **Spry1 cellular localization is regulated by the activation of MAPK, PI3K mediated by serum.** MDA-MB-231 cells were grown in poly-D-lysine coated coverslips in 10% FBS-aMEM growth medium. Twenty-four hours prior to immunofluorescence analysis, cells were either replaced with fresh growth medium (GM), starved (SF), or treated with 10mM U0126 (GM+U0126) or Ly294002 (GM+Ly294002), the MAPK or PI3K chemical inhibitor, respectively. Fixed cells were subjected for immunofluorescence staining of Spry1 and then F-actin staining using rhodamine-phalloidin. Image were obtained using Leica confocal microscope. The results starvation increases the cytoplasmic distribution of Spry1, and inhibition of MAPK and PI3K either decreases or increased cytoplasmic distribution of Spry1, respectively.

Supplementary Figure S10

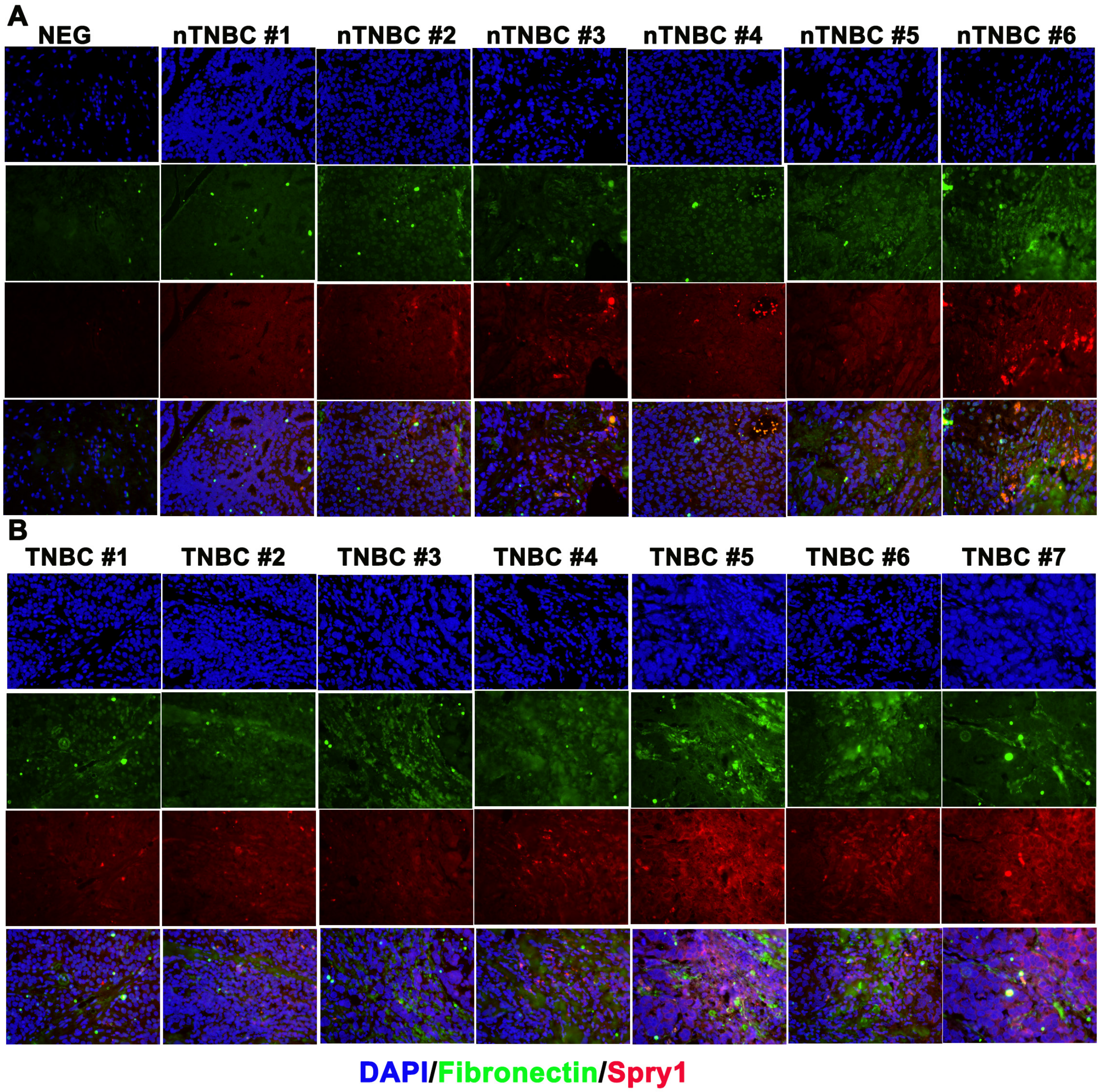

Supplementary Fig. S10. The increased Fibronectin expression is concomitant with higher Spry1 level in breast cancer tissues. Co-immunofluorescence staining of Spry1 (red) and Fibronectin (green) were performed on our available breast cancer array. Fibronectin expression is normally observed in extracellular matrix, and varied up-regulation of Fibronectin was observed in some of TNBC and non-TNBC (nTNBC) cells, and is concomitant with higher Spry1 expression. Images were obtained using Leica DM IRB.

Supplementary Figure S11

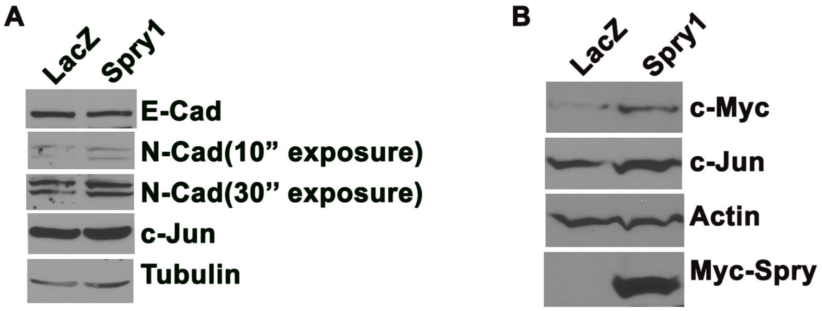

Supplementary Fig. S11. Overexpression of Spry1 in MCF-7 cells increases some mesenchymal markers expression. MCF-7 cells were transduced with myc-tagged mouse Spry1 for 48 hours. Cell lysates were subjected for immunoblotting assay using E-Cad, N-Cad, c-Jun, and c-Myc antibodies. The results show that overexpression of Spry1 slightly decreased E-Cadherin (E-Cad) expression, and slightly increased N-Cadherin (N-Cad), c-Jun and c-Myc expression.

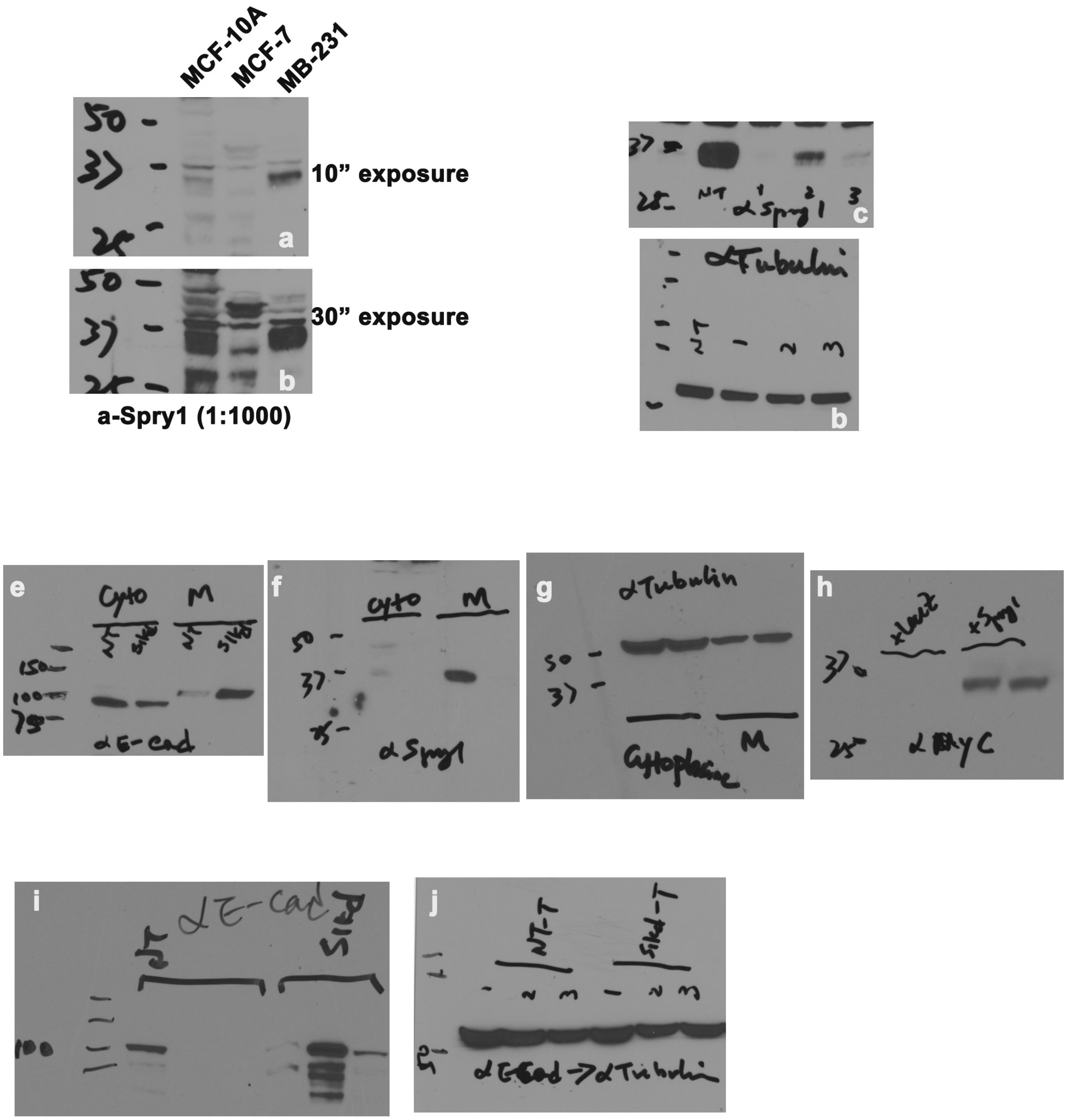

Supplementary Figure S10. Orignial immunoblotting results. a and b for Figure 1C. c and d for Figure 2A. e, f, g and h for Figure 4E. i and j for Figure 4H.

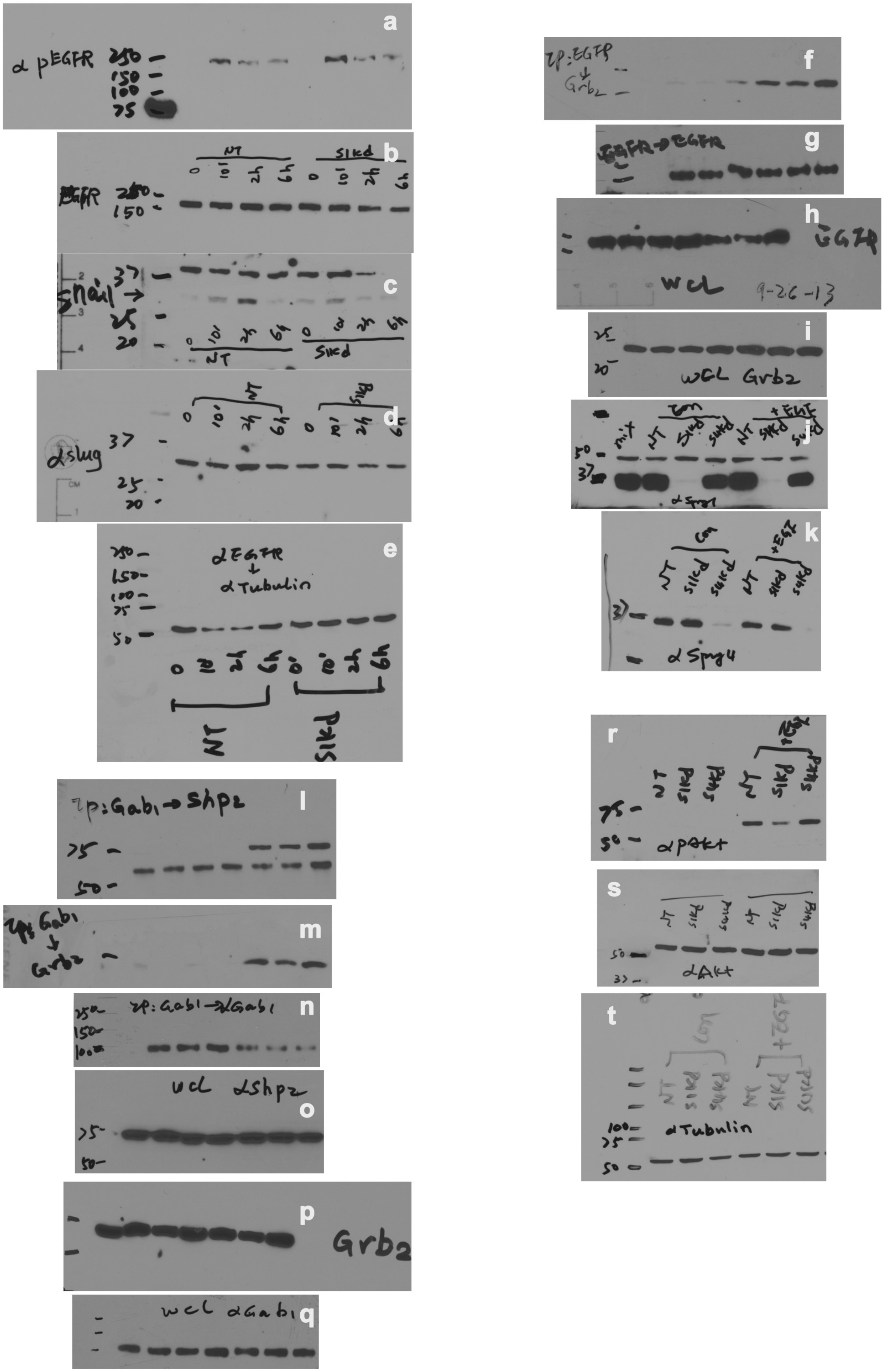
a
b
c
d
e
f
g
h
i
j
k
l
m
n
o
p
q
r
s
t
α pEGFR
αEGFR
αTubulin
Grb2
WCL Grb2
9-26-13
αSpry4
αpAkt
αAkt

Supplementary Figure S11. Orignial immunoblotting results. a, b, c, d and e for Figure 5A. f, g, h, i, j and k for Figure 5C. l, m, n, o, p and q for Figure 5E. j, k, r, s and t for Figure 5G.

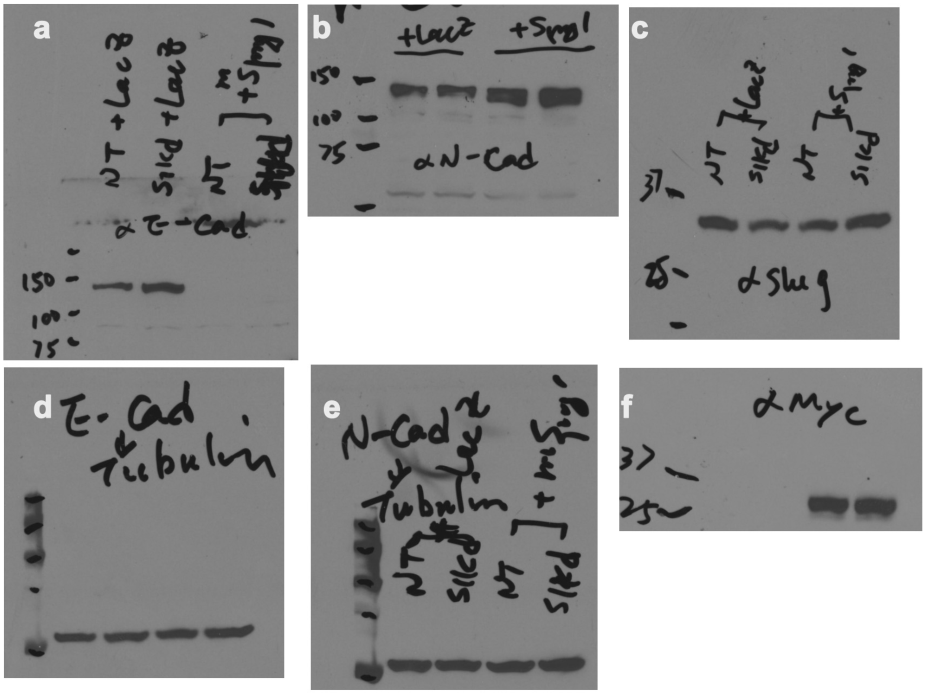

Supplementary Figure S12. Orignial immunoblotting results for Figure 6D.
